# When prevention science meets politics: a case study of macrosystem barriers to school-based CSEC prevention

**DOI:** 10.3389/fpsyt.2026.1876001

**Published:** 2026-07-17

**Authors:** Rochelle L. Dalla, Katie M. Edwards, Jennifer Stalder, Stacie Nessa, Aubrey K. Paschal, Laura Wolter, Darya Adams, Stephanie Olson, Lorey Wheeler, Layla Farhan

**Affiliations:** 1 Department of Child, Youth and Family Studies, The University of Nebraska-Lincoln, Lincoln, NE, United States; 2 School of Social Work, The University of Michigan, Ann Arbor, MI, United States; 3 Target high school liaisons, Anonymized high schools, United States; 4 The Set Me Free Foundation™, Omaha, NE, United States

**Keywords:** commercial sexual exploitation of children (CSEC), community-based participatory research, ecological systems theory, implementation barriers, macrosystem, prevention, school-based intervention

## Abstract

**Background:**

The commercial sexual exploitation of children (CSEC) is a critical public health issue in the United States, yet rigorous evaluations of primary prevention programs remain scarce. While ecological systems theory highlights the importance of macrosystem influences (e.g., policy, political climate), these factors are rarely examined as direct barriers to prevention implementation and evaluation.

**Objective:**

This paper describes a community-based participatory research (CBPR) case study of Project LIVE—a federally funded, school-based CSEC prevention trial in a public school district in a midsized Midwestern city—to examine how macrosystem-level forces disrupted program implementation and compromised the feasibility of rigorous evaluation.

**Methods:**

Drawing on a multi-year (2022–2027) CDC-funded initiative, we synthesize recruitment and retention data, implementation records, and contextual documentation across an open pilot trial and a planned quasi-experimental design. Using an ecological systems framework, we analyze how federal, state, and district-level policies and events shaped study outcomes.

**Results:**

Despite strong community partnerships, high program relevance, and extensive recruitment efforts, enrollment and retention were insufficient to support planned analyses (e.g., <13% post-test completion in the quasi-experimental phase). Key barriers included a state-mandated shift from passive to active guardian consent, federal immigration enforcement policies that heightened fear among immigrant communities, mass federal workforce reductions affecting grant oversight, and substantial school district administrative instability. These macrosystem disruptions cascaded across micro, meso, and exosystems, ultimately leading to early termination of programming.

**Conclusions:**

Findings underscore the critical yet underexamined role of macrosystem forces in shaping the feasibility and success of school-based prevention research. Even well-funded, theoretically grounded, and community-engaged interventions may fail under conditions of political instability and policy misalignment. Future prevention science should incorporate assessments of policy context, community readiness, and structural risk, and prioritize flexible, adaptive designs capable of withstanding macro-level disruptions.

## Introduction

On January 22, 2026, the Governor of Iowa publicly reaffirmed the state’s dedication to fighting human trafficking by signing a proclamation declaring January as Slavery and Human Trafficking Prevention month. Simultaneously, Project LIVE, a CDC-funded project aimed at evaluating the efficacy of a school-based human trafficking prevention program offered in the target Midwestern city’s public high schools (hereafter PHS), received an Outstanding Anti-Trafficking Service Award^1^. Despite expectations of curriculum delivery and a rigorous clinical trial evaluation involving over 7,000 students, Project LIVE has been continuously stymied by federal and state policies—those representing the macro-system in ecological systems theory (EST, 1, 2). Using PHS and Project LIVE as a case study, this paper examines the intersections of anti-trafficking efforts, target populations primed for impact, and current U.S. policies which deeply constrained efforts to meet funding mandates–despite public-facing awards and declarations to the contrary.

## CSEC vulnerability and risk

The commercial sexual exploitation of children (CSEC), which includes sex trafficking, is a public health crisis in the United States (U.S.) (3, 4). CSEC refers to a “range of crimes and activities involving the sexual abuse or exploitation of a child for the financial benefit of any person or in exchange for anything of value (including monetary and non-monetary benefits) given or received by any person,” (5). Sex trafficking, a form of CSEC, is defined as “the recruitment, harboring, transportation, provision, obtaining, patronizing, or soliciting of a minor for the purpose of a commercial sex act,” (6). CSEC constitutes an adverse childhood experience and often co-occurs with other forms of violence such as physical assault and psychological abuse (7).

Theory-based research on vulnerability factors that increase risk for CSEC is critical for creating effective programming. Systems approaches, which allow for examination of intersecting vulnerabilities across the entire socio-cultural context, are particularly useful for identifying risk as well as understanding how youth are recruited, groomed, and coerced into trafficking situations (8). Ecological systems theory (EST; 1, 2, 9) provides a relevant and practical framework from which to examine myriad and multi-layered risk associated with CSEC and the processes through which trafficking occurs across contexts. Bronfenbrenner argued that developmental processes cannot be understood without careful observation of the entire ecological context in which an individual is embedded. EST positions the developing person at the center—recognizing that each person’s unique characteristics (including personality, physicality, mannerisms, reactions) impact how that person interacts with their social environment as well as how, at least to some degree, the social environment interacts with the developing person.

*Individual-level* risk factors for CSEC include victimization by other forms of child abuse and maltreatment (e.g., physical abuse, neglect), being a runaway and/or homeless, being system-involved, and having low self-worth/self-esteem. Sexual and gender minority youth, youth with disabilities, youth of color (e.g., Black youth, Indigenous youth), and youth living in poverty are also at increased risk for CSE and are disproportionately targeted by traffickers due to structural vulnerabilities and reduced access to protective resources (10–13). Although some research suggests that girls may be at higher risk for CSE than boys, more recent research suggests this may not be the case particularly within sex trafficking contexts where boys are less likely to be identified or reported and may experience exploitation through distinct pathways (e.g., online recruitment, survival sex, or in instances where labor and sexual exploitation overlap) (14).

The microsystem in EST refers to social systems that contain the developing person. Among typical U.S.-based youth, these often include home/family, school, and peer groups. Additional microsystems include church groups, employment, and/or other types of social systems in which a youth is embedded (e.g., volunteer organization). The mesosystem refers to developmental processes *across and between* microsystems. The bulk of research examining CSEC-risk is concentrated at the micro and mesosystem levels. These factors include, for instance, parent/caregiver dysfunction (e.g., addiction, mental health problems, criminal behavior), involvement with peers who engage in the sex economy, including survival sex, peer and/or school systems which normalize gender-based violence and/or the objectification or glamorization of a pimp culture, all of which can facilitate grooming, recruitment, and normalization of trafficking dynamics among youth (15, 16).

The exosystem refers to neighborhoods and community contexts in which the developing person is embedded. Unlike micro and mesosystems, the exosystem exerts influence somewhat *indirectly* via its influence on the relational dynamics and processes of groups within those neighborhoods and communities. CSEC risk at the exosystem level includes living in neighborhoods and environments characterized by crime, poverty, community-based violence, as well as street-based sex and drug economies that create conditions in which trafficking networks can operate and in which youth can be more easily exploited (17–20).

Bronfenbrenner describes the macrosystem as the “blueprint” of society. The macrosystem is considered the most “upstream” system in the EST model as it sets the tone for how societies operate. The macrosystem includes, among other things, political and economic structures of a society, as well as legal systems and taboos that impact relational dynamics and social processes in “downstream” socio-cultural environments including how sex trafficking is defined, detected, prevented, and responded to across systems.

EST has been applied previously in the examination of child sexual abuse, sexual violence, and human trafficking. For instance, Martinello (21) examined factors related to child sexual abuse through an ecological systems lens (i.e., risk at the micro, meso, exo, and macrosystem levels). Similarly, Stockman et al. (22) applied an ecological systems approach to understanding the impact of sexual violence via a systematic meta-review. In a recent study, Twis et al. (23) examined correlates of domestic minor sex trafficking at the exo and macrosystemic levels specifically (e.g., digital environment, community ties, anti-trafficking education and legislation). However, examination of macrosystemic influences related to CSEC prevention is atypical. And, aside from Twis et al. (23) the macrosystem is often given a “passing glance” only—with research focused instead on those areas of the ecology where direct intervention and impact can be more easily obtained. This paper is different. Whereas the macrosystem is understood through its downstream effects on the individual (providing indirect influence through the micro, meso, and exosystems), here we describe instead how Project LIVE, a five-year federally-funded program to help reduce CSEC among highschoolers—and the youth intent on receiving the curriculum, has been consistently *derailed* by macrolevel influences—particularly those related to politics and political decision-making at the federal, state, and local school district levels.

### CSEC intervention

Although there are growing efforts to address CSEC and sex trafficking, the majority of these efforts remain focused on downstream responses, including criminal prosecution of traffickers and services for survivors after exploitation has occurred (19, 24). Far less attention—and substantially less rigorous evidence—has been devoted to primary prevention, particularly among youth prior to exploitation. Existing frameworks emphasize that effective prevention must operate across multiple domains, including reducing perpetration, strengthening bystander intervention, and equipping youth with skills to recognize and resist recruitment and grooming tactics. School-based programming represents a particularly promising setting for such efforts, given its reach among youth at elevated risk for trafficking and its potential to shift peer norms, increase awareness of recruitment strategies, and build protective skills.

Despite this promise, the evidence base for youth-focused CSEC prevention remains limited. Many existing programs are awareness-based, target narrow populations, or focus primarily on victimization risk rather than the broader dynamics of trafficking, including perpetration and bystander engagement (25–27). Critically, few programs have been evaluated using rigorous designs, and those that have are often limited by small samples, single-group designs, or internally conducted evaluations with limited dissemination (25, 28). As a result, there is a substantial gap in understanding not only what works, but whether rigorous, school-based evaluations of trafficking prevention programs can be successfully implemented in real-world settings. This gap is particularly important given increasing federal and community investment in prevention programming, as well as expectations that such programs be evaluated using quasi-experimental or randomized designs. Project LIVE was designed to address this gap through a rigorous, school-based prevention program.

## Case study

As a case study (29), this paper focuses exclusively on attempts to evaluate a CSEC prevention curriculum, utilizing community based participatory research (CBPR) methods, among high school youth in a single school district (PHS) in a mid-sized, midwestern city. Attention is paid to macrosystem influences that continuously derailed efforts intended to protect youth by mitigating gender-based violence and sexual exploitation. In the pages that follow, we first summarize the federal grant which supported the project and then describe the demographic context of the target population, followed by a description of the methods used to achieve benchmarks and funding deliverables. We finish with a detailed description of the systemic macrolevel processes that resulted, ultimately, in winding down study activities early without meeting all study aims.

### Target site

Despite its Midwestern geography, the capital city in our target state, and PHS specifically, are both demographically *unrepresentative* of the rest of the state. According to the most recent Census data (2022), the target city is more ethnically and racially diverse (16.3% Latinx/Hispanic; 11% Black) and more impoverished (15% poverty rate) than the state (i.e., 7.9% Latino/Hispanic, 4% Black, 11% poverty rate) (30). The city also far exceeds the remainder of the state in its refugee and immigrant populations (14.2% vs. 5.9%, respectively) (30). Comparatively, 20% of the U.S. population is Latinx/Hispanic, 12% is Black, and 10% is impoverished; 14.1% of the U.S. population consists of immigrants and refugees. Alarmingly, according to the 2024 FBI Uniform Crime Report data the violent crime rate (e.g., murder, rape) in the target city is 896% times greater than the national average, with a safety grade of F (indicating high risk) (31).

Des Moines’ PHS consists of more than 60 schools with a population of approximately 30,000 students. PHS serves about 8,500 students in five traditional high schools and three “alternative” high schools. The 8,500+ student population is extremely diverse, with youth representing 88 nationalities and whose households speak nearly 100 different languages. Sixty-five percent of PHS youth are from racial/ethnic minority groups and 75% receive free or reduced lunch. PHS school personnel closely match regional demographics consisting of majority White, non-Latino persons. Three of the traditional high schools have chronic absenteeism rates exceeding 50%; with chronic absenteeism across all PHS secondary schools ranging from 20% to 73% (32). PHS high school students reflect many risk factors for CSEC (e.g., racial/ethnic minoritized status, low income, homelessness, in addition to chronic absenteeism) and are an ideal target population for CSEC prevention programming.

## Project LIVE

### The grant: brief overview

**Figure 1:**
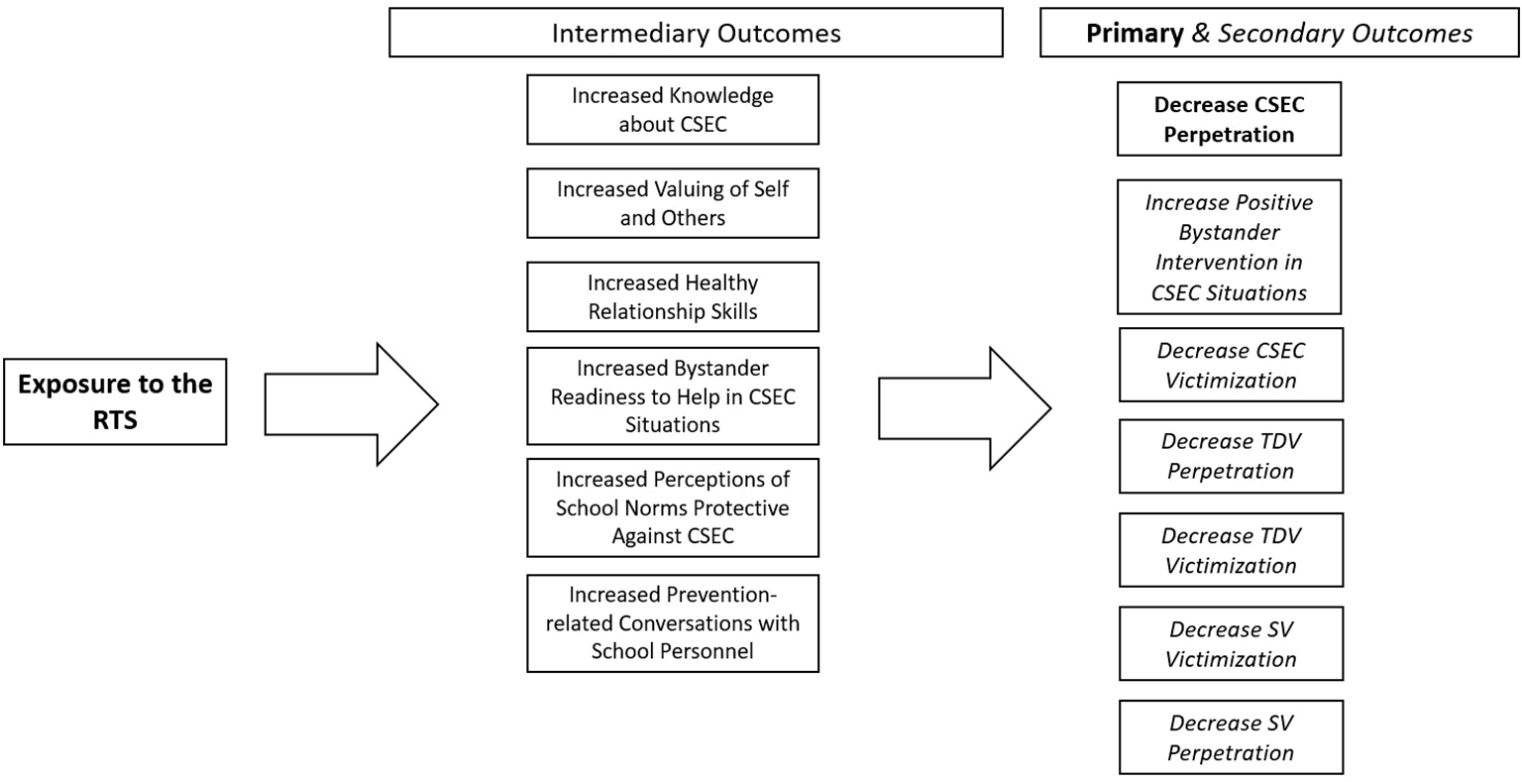
Anticipated outcomes following exposure to RTS curriculum.

We were awarded a five-year (2022-2027) CDC-funded grant intended to evaluate a school-based CSEC prevention program among high-risk, high school students. Three main collaborators were involved: the research team who conducted the evaluation, the Set Me Free Project (SMFP) who created and delivered the curriculum (READY to Stand or RTS)^2^, and the target school district. Funding occurred in two phases (components A and B), with a mandatory re-submission after component A. Component A (2022-2023, grant years one and two) included refinement, planning, and pilot. In these formative years, we conducted focus groups with students and school personnel, enhanced the RTS curriculum to include bystander readiness and school culture components, and conducted an open pilot trial (OPT).^3^ The OPT included two high schools (one traditional, one alternative; see below). Students received six 45-minute modules of programming, and completed a pre- and post-test, as well as post-session surveys (six), and six-, 12-, and 18-month follow-up surveys. **Figure 1** provides a snapshot of the anticipated intermediary, primary, and secondary outcomes associated with RTS exposure. We estimated a sample size of 879 students (90% of those eligible).

**Figure 2:**
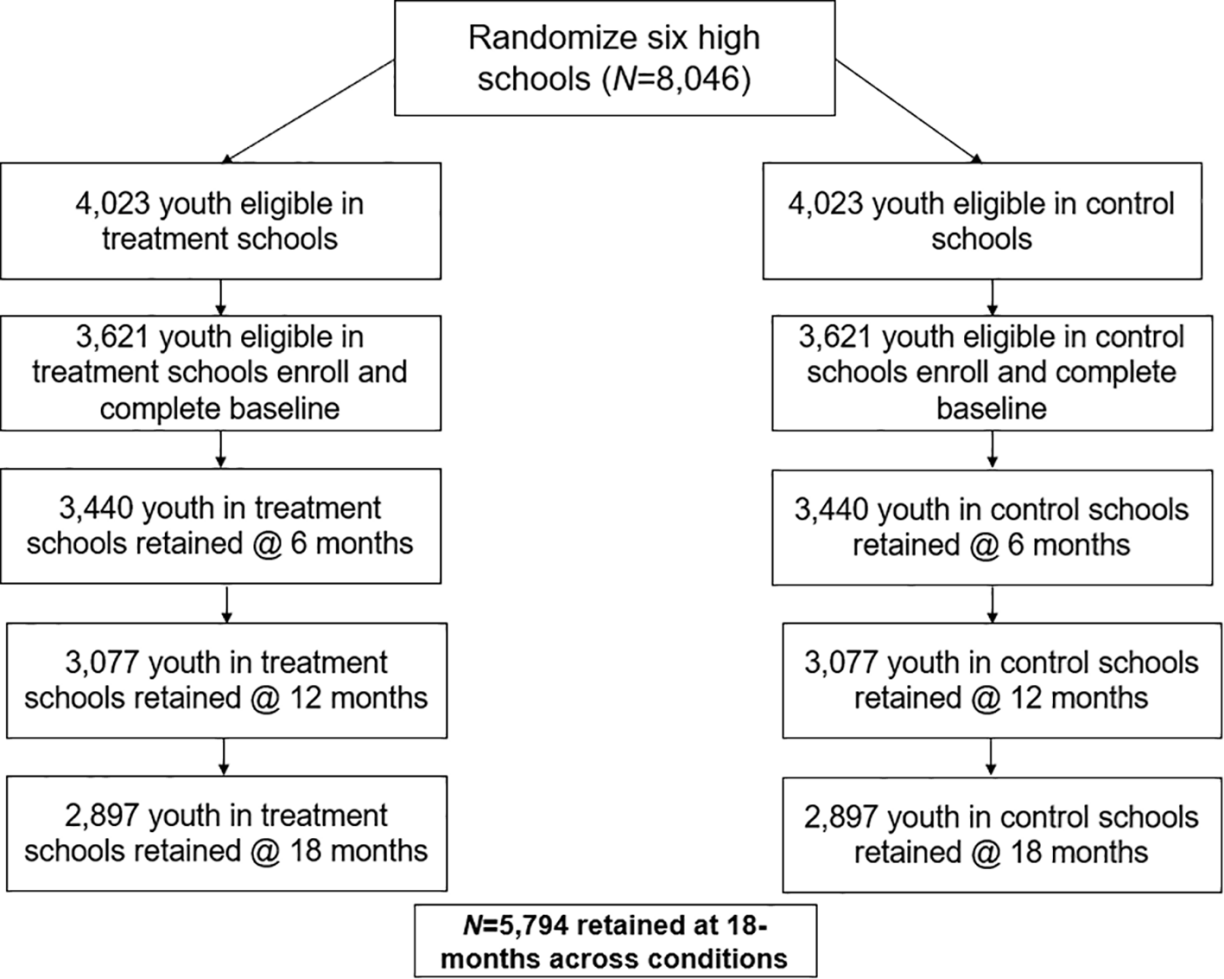
Consort chart for component B, quasi-experimental design (*Anticipated*).

Component B (2024-2027, grant years three to five) included the quasi-experimental design (QED) and rigorous evaluation of the RTS curriculum. Six schools were targeted (four traditional and two alternative), not including those involved in the OPT. Three schools were targeted to receive the programming (treatment) and three were to serve as waitlist controls (delayed programming until all surveys were completed). Survey administration mirrored that of the OPT. **Figure 2** presents a consort chart of anticipated sample size across both treatment and control conditions.

## Challenges to achieving benchmarks and deliverables

### Pilot (grant years 1 and 2, 2022–2023)

**Figure 3:**
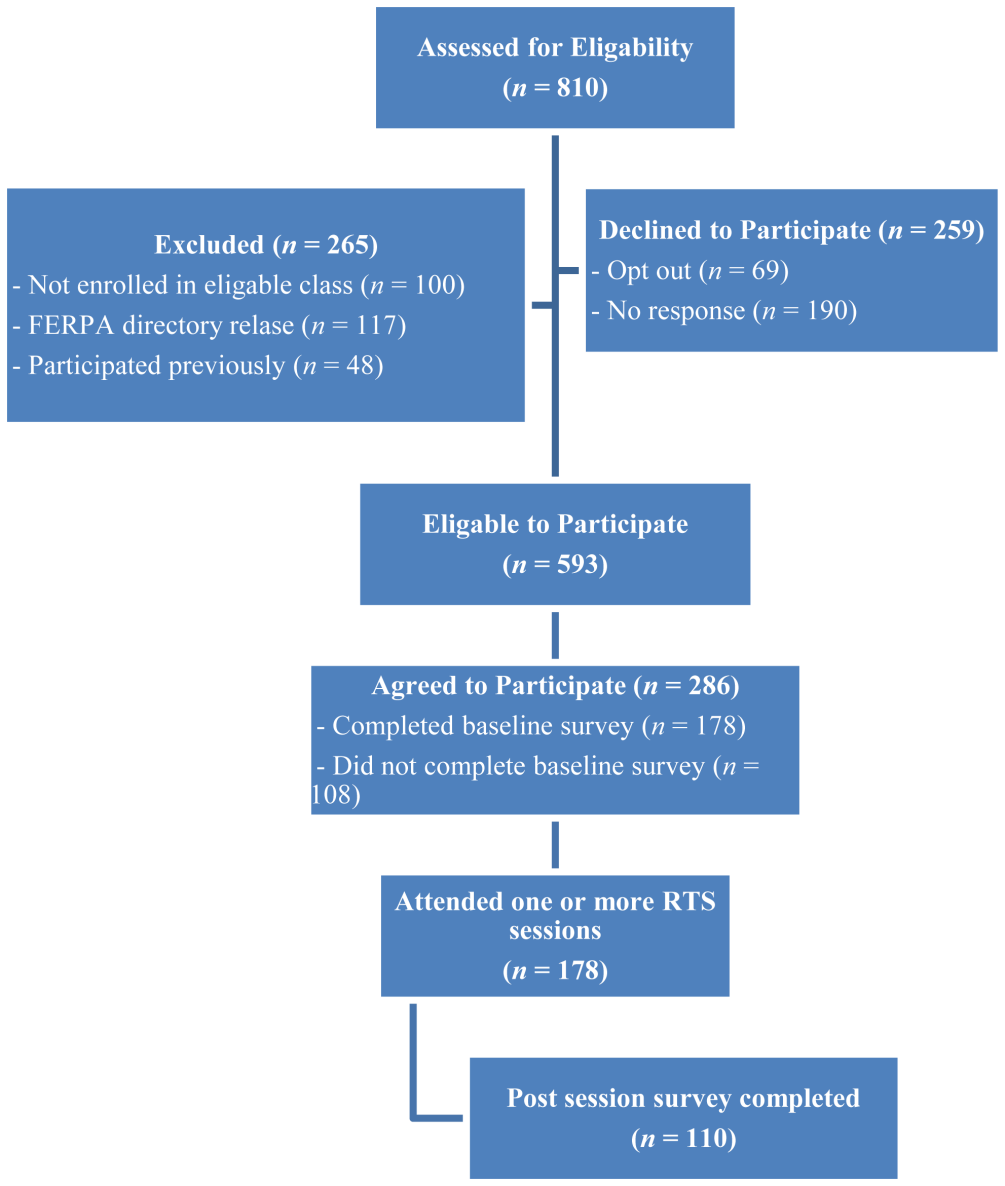
consort chart for Component A, open pilot trial *(Reality)*.

Although focus groups and program enhancement goals were successful, unanticipated challenges derailed our ability to successfully achieve stated benchmarks and deliverable from the OPT. The biggest challenge was in recruitment—reaching a sample size that could withstand complex statistical analyses of outcome variables across two groups. Despite labor-intensive recruitment strategies, programming served far fewer students (*n* = 178 completing pre-test; *n* = 110 completing post-test; refer to **Figure 3**) than the anticipated number of 878.

Challenges and roadblocks to the OPT are detailed in an earlier paper (36). Summarized here, the biggest challenge was a state-issued bill mandating guardian *active-consent* for school-based programming with minors (students ≤ 17 years of age). The legal change took effect in the fall of 2023—the same as the target implementation of the OPT. At the time the grant was submitted and funded, Iowa was a passive-consent state for school-based programming. We had little time (programming was beginning in fewer than three months), and no dedicated funding (as the budget had already been approved) to adequately attempt active guardian consent. To pivot quickly, we first pushed programming out until spring of 2024 and then focused exclusively on students in elective classes as parents would be unlikely to allow students to miss “core” courses; we focused specifically on gym and health classes. Student recruitment then involved: vising all eligible classrooms (across eight periods) on multiple occasions, explaining the study, and handing out guardian consent forms (in multiple languages), making hundreds of telephone calls and sending equal numbers of text and email messages to parents/guardians with students in the target classes, in addition to canvasing neighborhoods where students in eligible classes lived in order to talk to guardians directly to describe the study and its potential impact. The labor-intensive house-to-house visits were our best form for recruiting. In addition to recruitment challenges, we unexpectedly encountered problems related to “student attrition” when moving students from their health/gym class to the dedicated classroom where programming was delivered. That is, students simply left; we had no authority to stop them from leaving the school after they left their classes.

### QED (grant years 3–5, 2024–2027)

We gained much knowledge from the OPT and made several “corrections” that, we were certain, would ensure entirely different outcomes in the QED with regard to student recruitment and retention, and guardian consent. Corrections included: (1) expanding our target elective classes (to include psychology, sociology, child development, and any others that principals would approve, *in addition* to health and PE classes); (2) adding study recruitment materials in school registration packets (it was too late to do this in the OPT by the time the law changed); (3) using hall monitors to assist in moving students from their classroom locations to the programming sites to enhance student retention during the walk; and (4) initiating a RedCap-based matching system to track students across time points. In addition, we continued to call, email, and text guardians of students in all eligible classes, in all six high schools, as well as work with student and adult advisory boards and our PHS liaisons to generate additional ideas (e.g., attending community events and school functions) to explain and then disseminate consent forms—which we did.

These efforts were to no avail. That is, despite monumental, labor-intensive work, recruitment response rates (returned consent forms and students physically present to receive programming) were miserably low. Of 5,810 eligible students (across treatment and control schools), 280 were ultimately enrolled (*n* = 92 treatment; *n* = 188 waitlist control). The percentage of T1 (pre-test) survey completers who also completed T2 (post-test) was only 12.9% across both conditions. We simply did not have a large enough sample to conduct necessary analyses^4^.

That said, continuing Project LIVE in this context would compromise statistical power and internal validity, limit the ability to rigorously evaluate program effectiveness, and reduce the overall contribution of the project to CDC’s prevention science portfolio. In late April 2026, we made the very difficult decision to stop CSEC prevention programming in PHS, despite having one year of funding remaining.

## Macrosystem influences and a perfect storm

As noted earlier, the macrosystem refers to the larger socio-cultural context that creates norms, values, and processes for how things work in a society. Political systems are macrosystems. Three primary political macrosystems (federal, state, and PHS system) exerted tremendous influence on the implementation of Project LIVE and achieving grant-based objectives. Macrosystem events, issues, and processes with direct impact on Project LIVE are summarized in **Table 1**.

**Table 1:** Macrosystem events, issues, & processes impacting Project LIVE.

Macrosystem	Event, issue or process	Impact on Project LIVE
Federal Government	ICE Presence and Deportations	• Cancellation of door-to-door recruitment; • Guardians unlikely to sign any document • Increased student absenteeism
	Grants and Funding Agencies: • Mass firing of CDC Employees • Extermination of hundreds of federally funded grants	• Project LIVE consultants fired; • Uncertainty about funding status or future programming • Principal investigators (PIs) focus shifts to fall out (e.g., suing federal government, retaining staff, finding alternative ways to run community-based programs).
	DOJ investigations of PHS hiring practices	• PHS school culture of fear and uncertainty; low morale among students and school personnel
State Government	Senate File 496: Education, Parental Rights, and School Transparency (May, 2023)	• Immediate shift from passive to active guardian consent for student participation
PHS	Firing of Superintendent	• PHS culture: fear, uncertainty, low morale, trauma
	Administrator Turnover	• Shifting administrator, lack of role clarity, limited capacity to take on new roles (e.g., necessitated by Project LIVE and school-based recruitment, survey completion and programming)

### Federal

Factors at the federal level (e.g. anti-immigration policies, deployment of Immigration and Customs Enforcement (ICE) agents, and mass-firing of CDC staff are directly implicated in programmatic complications).

ICE. Trump’s second term stated campaign promised to execute “the largest domestic deportation operation” in U.S. history—with a goal of deporting one million people during his first year (37). This priority is funded by the One Big Beautiful Bill Act which allocates $170 billion to enforcement (38). These tactics, push the limits of presidential power and include expedited deportations, deporting individuals without cause—sometimes to third world countries where they have no ties—amplified immigration raids in businesses and communities, and granting of new powers—seemingly without proper congressional approval—to federal, state, and local officials to enforce immigration laws (38). The January 2025 ICE killing of two U.S. citizens sparked mass backlash—but immigrant communities across the country had already been severely damaged (39). According to the *Migration Navigator* (40): “ICE Deportation involves the detention and expulsion of individuals found to be unlawfully present in the United States. The process is complex, impacting not only the individuals deported but also their families and communities, often resulting in long-term psychological, economic, and social repercussions. The emotional toll on families, particularly children who may be US citizens, is profound”.

In Iowa, specifically, immigration arrests have increased by 276% since President Donald Trump took office compared with rates in 2024 (41). Further, in October, 2025 an 18-year-old PHS student was detained and deported by ICE. After being transferred to a state jail, the student was sent to a detention center in Louisiana before being deported to a Central American country (42).

To be very clear, the target school district includes a substantial number of refugee, migrant and immigrant families, many of whom were directly impacted by heightened federal immigration enforcement. The fear of federal agents and deportation—particularly in the context of widely publicized detainments and uncertainty about legal protections was, and continues to be, palpable across the community. This climate of fear had immediate and tangible implications for Project LIVE. Most notably, any and all plans for door-to-door recruitment—our most effective strategy for obtaining guardian consent during the OPT—were cancelled immediately due to safety concerns for both families and research staff.

Further, although we cannot establish causality, we strongly suspect that these federal policies and the broader climate of fear and trauma they produced significantly affected parents’ and guardians’ willingness to engage with the study. Specifically, reluctance to sign formal documents, such as consent forms, may have been heightened by concerns about surveillance, documentation, or unintended exposure to authorities. In this context, standard research practices (e.g., written consent) may have been perceived as risky rather than routine, thereby directly undermining recruitment and participation.

CDC staff and grant exterminations. In early 2025, and under Trump administration mandates, health secretary Robert F. Kennedy Jr. initiated wide-sweeping layoffs and position eliminations to the Department of Health and Human Services (DHHS; 43). At the CDC 1,300 staff (or 10% of the workforce) were removed. Executive Director of the American Public Health Association, Dr. Georges Benjamin, referred to the CDC cuts as, “indiscriminate, poorly-thought out layoffs [that would be] very destructive to the core infrastructure of public health” (43). The CDC was not alone. As many as 1,500 employees at the National Institutes of Health (NIH) were also laid off. The layoffs at the CDC impacted Project LIVE *directly.* Project LIVE is funded as a cooperative agreement through the CDC’s National Center for Injury Prevention and Control (RFA-CE-22-003). Most of our CDC partner team—a team that met regularly with Project LIVE researchers and SMFP staff, a team who conducted a site visit to the target city in July of 2023 and met our Youth and Adult Advisory Boards, a team who offered guidance and advice, and who oversaw all grant-mandated initiatives, was fired.

Trump’s vision of radically reshaping the landscape of federal research funding was acted upon with impunity in the early months of his second administration—with sweeping cuts, cancellations and disruptions to programs perceived as promoting “gender ideology,” “radical indoctrination” and DEI (44). In most cases, principal investigators (PIs) were sent “non-continuation” letters indicating that the research supported by the grant, for example, no longer “…effectuates the agency’s needs and priorities…” (45).

Surprisingly, Project LIVE was not eliminated during those expansive cuts. However, the Project LIVE PI suffered the elimination of nine grants (seven from NIH alone) totaling more than $14 million. The PI’s immediate priorities were, as they should have been, retaining her staff of more than 50 and finding alternative ways to maintain those programs. Those concurrent demands required redistribution of leadership responsibilities across the Project LIVE research team, resulting in brief periods of transition as team members assumed new roles. However, it is important to note that these challenges unfolded alongside broader macrosystem disruptions, which independently and substantially constrained Project LIVE implementation.

DOJ investigates PHS. On September 30 of 2025, PHS became officially under investigation by the Department of Justice’s (DOJ) Civil Rights Division regarding the district’s employment practices. As reported by the Office of Public Affairs, the Justice Department received information that PHS may be engaged in employment practices that discriminate against employees, job applicants, and training program participants based on race, color, and national origin—in violation of Title VII. DEI initiatives and race-based hiring preferences violate federal anti-discrimination laws. Assistant Attorney General Harmeet K. Dhillon commented, “School districts must cease these unlawful programs and restore merit-based employment practices for the benefit of both students and employees” (46).

### Iowa

Senate File 496: Education, Parental Rights, and School Transparency. Signed into law May 26, 2023, this bill, otherwise known as the “Parental Rights Bill”, made a number of changes to the state’s school operations, including school curriculum, school transparency, and special education services (47). With direct and immediate repercussions on Project LIVE, this Bill “…requires school districts, charter schools, and innovation zone schools to obtain prior written consent of a student’s parent or guardian before requiring a student to participate in any survey or formal assessment….School districts are also prohibited from releasing information about students without receiving parental consent.”

With this Bill, programming without parental consent was impossible. Massive recruitment initiatives were introduced into the project, at the cost of tremendous financial and labor resources, that had not previously been warranted, planned for, or budgeted. As described earlier, enrollment numbers estimated in the thousands dwindled to hundreds.

### PHS

Rather than addressing the entire PHS structure, we focus instead on the PHS structures and administrative roles with direct implications for Project LIVE programming. This consists largely of our PHS liaisons (JS and SN) and their supervisors. The research team worked directly with the liaisons—meeting weekly or more often as necessary—who then worked with their supervisors as needed to ensure smooth operation of the Project. The liaison supervisors (and their supervisors) were frequently needed to (1) approve research team requests (e.g., access to guardian contact information and class rosters), (2) provide non-monetary resources (e.g., space for programming), and (3) move requests further upstream (to those with greater authority), as needed. Critical “asks” of the liaisons included, for instance, including recruitment materials in school registration packets, working with principals to identify “eligible” elective classes from which to pull students, getting classroom rosters for those classes, providing space for programming and survey administration, and assigning hall monitors to accompany students from classrooms to programming sites, etc … In short, the liaisons provided critical connective tissue across and between the research team and the implementation sites (i.e., the eight high schools in PHS).

ICE detainment of PHS superintendent. Administrative changes often create (usually temporary) chaos and uncertainty; learning new systems and adapting to new roles and responsibilities can take time. This is understandable. However, in the case of Project LIVE, the administrative changes were not only extensive but also rapid, unpredictable, and sometimes shrouded in secrecy thereby creating a school climate characterized by angst, fear and tension. Most notably was in fall, 2025 when the District Superintendent (Dr. X) was detained by ICE for failing to comply with a final order of removal by an immigration judge. Lacking any knowledge of Dr. X’s illegal immigration status, the highly publicized arrest hit residents hard with fears of racial profiling, on the one hand, and rumors of ICE taking control of communities, on the other. In a city with high concentrations of immigrants, the impacts were traumatic. According to JS (liaison to PHS, personal communication, April 17, 2026): “That day [September 26, 2025; day of Dr. X’s arrest] was the most traumatic day in our entire school district history … and then the trauma continued, and it planted doubt. Especially in central school government. There were significant impacts. It was a very polarizing time, as facts came out, among staff and students, and it divests attention from teaching, and supporting…”

The Iowa Board of Educational Examiners stripped Dr. X of his professional administrator’s license and the PHS School Board has subsequently filed a law against the search firm which identified Dr. X as a suitable candidate for the superintendent position. Not surprisingly, this highly publicized case led immediately to the DOJ’s investigation of PHS hiring practices (noted earlier).

PHS administrative turnover. The fall of 2025 mark a significant turning point in, and rapid acceleration of, PHS administrative turnover. Summarizing **Table 2**, there have been four superintendent changes (and three different superintendents) since Project LIVE began and our district liaisons have had four different supervisory changes since April of 2024, with the Director of School Climate and Culture position eliminated (without cause). Further, and not reflected in **Table 2**, is that the High School Director was fired (Jan. 2024) and subsequent principal turnover occurred in four of the eight high schools in which Project LIVE was involved; the remaining four have had turnover in their Associate Principal teams. Given the scope and pace of these changes, it would be foolish to assume that Project LIVE implementation was ***not*** affected.

**Table 2:** PHS administrative changes.

Decision-making & supervision Hierarchy	AY 2021-2022 grant submission		AY 22-23 grant Y1 (open pilot trial, OPT)	AY 23-24 grant Y2 (OPT)	AY 24-25 grant Y3 (Quasi-experimental design, QED)	AY 25-26 grant Y4 (QED)	AY26-27 grant Y5 (QED)
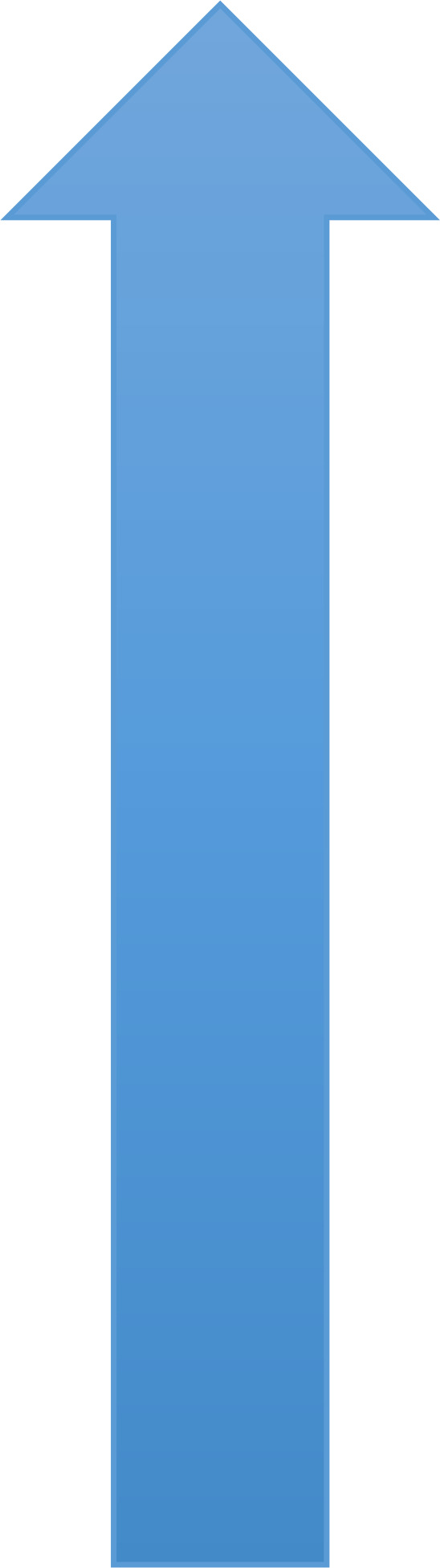	Superintendent	Super A^1^	Super B^2^	Super C (Dr. X)^3^	Super C	Super C^7^ (Sept.25’) then ➔ Super B (again)	Super B^9^
	Executive Director of Student Services	ED	ED	ED	SN➔AA	SN➔AA	SN➔New Hire (planned for July 1, 2026)
					JS➔ED	JS➔AS^5^ (Oct., 25’) then➔CO^6^	JS➔New Hire^8^ (planned for July 1, 2026)
	Director of School Climate and Culture	CC	CC	CC	Position eliminated		
	Project LIVE Liaisons	JS & SN	JS & SN	JS & SN	JS & SN	JS & SN	JS & SN

^1^ Wrote original letter of support for grant.

^2^ Interim position; former superintendent did not finish out contract.

^3^ Wrote second letter of support; grant resubmission was required for final three-years of funding.

^4^ AA, Administrator of Alternative programs reports to RB, Chief of Operations.

^5^ AS, Associate Superintendent.

^6^ CO Chief of Operations.

^7^ Detained by U.S. Immigration and Control Enforcement (ICE) officers on charges of immigration violation.

^8^ New Hire, Executive Director of Federal Programs and Student Supports. If structure remains as it has, JS will report to this person.

^9^ Interim position through June 30, 2027.

Winding down grant activities. At the time of this submission, grant activities in PHS have ceased. Nonetheless, we are committed to preserving the scientific aims of the project and maximizing its broader contribution to prevention science. In this vein, and with one year of funding remaining, we have been given approval by and support from our CDC partners to identify alternative implementation sites with greater feasibility for program delivery and evaluation, while simultaneously reframing the context of Project LIVE as an implementation-case study of how macrosystem-level forces can disrupt prevention efforts. Rather than representing a departure from the original goals, this approach reflects an adaptation to real-world constraints and highlights the importance of understanding not only whether prevention programs work, but whether they can be implemented and evaluated under shifting policy and political conditions.

Although Project LIVE is no longer operational, relationships with our district liaisons remain strong and our weekly meetings—involving the research team, the SMFP, and our liaisons—continue. This is particularly relevant because, as noted above, the implementation-based case study requires continued engagement with PHS administrators, parents/guardians, students, and other community informants. In addition, new grant activities involving the liaisons as collaborators are being actively pursued.

## Summary thoughts and lessons for future programming

As a case study, the phenomenon detailed here is simultaneously heart-wrenching—given the clear need for and value of CSEC prevention among the target youth, striking in the magnitude of financial, labor, and temporal resources invested relative to the limited evaluable data obtained, and ultimately affirming of the extensive efforts undertaken by the research team, PHS liaisons, the SMFP, and Advisory Boards (i.e., micro, meso, and exosystem partners) to meet project benchmarks. Importantly, the majority of benchmarks were, in principle, achievable; however, macrosystem-level forces fundamentally constrained implementation and evaluation efforts. In this sense, it was not the project that failed, but the broader structural conditions necessary to support it. If nothing else, this case study should serve as a learning opportunity for like-minded social scientists intent upon helping youth—and their communities—become intolerant of all forms of violence, knowledgeable of CSEC risk, and skillful in resisting perpetrator efforts aimed at exploitation and harm.

## How did we get here?

**Figure 4:**
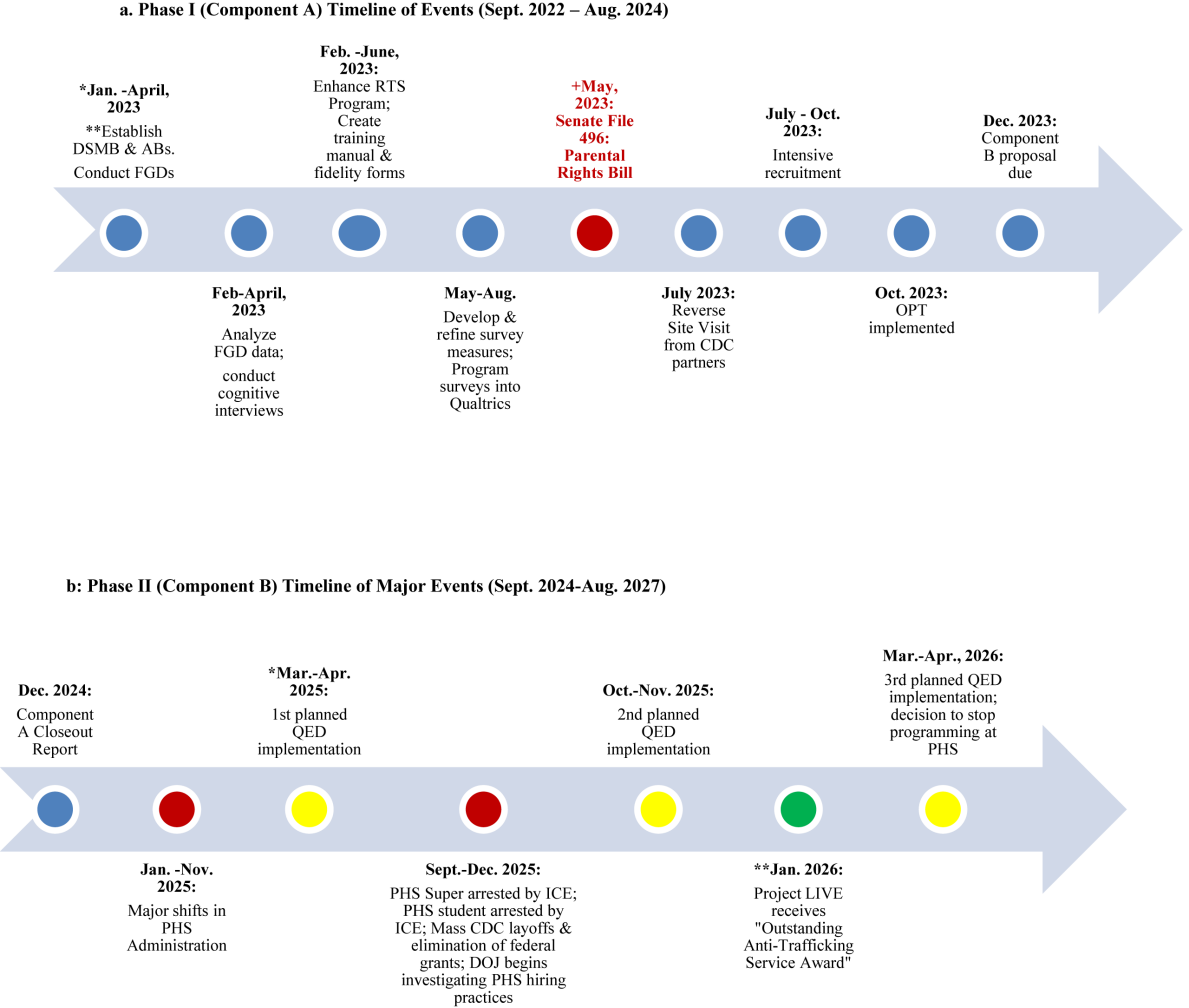
**(A)** Phase I (component A) timeline of events (Sept. 2022 – Aug. 2024) *Weekly meetings with CDC Partners throughout Component A **DSMB (Data and Safety Monitoring Board); AB (Advisory Boards; established with youth, guardians, and community stakeholders); FGD (Focus Group Discussions); +Red highlight: critical event/issue negatively impacting Project LIVE programming. **(B)**: Phase II (Component B) timeline of major events (Sept. 2024-Aug. 2027) *Yellow highlight: unable to move forward with implementation due to extremely low recruitment responses; final QED date (March-April, 2026 resulted in <5% response rate). **Green highlight: Positive recognition of Project LIVE *potential*.

A summary of critical events, as they unfolded throughout the life-course of Project LIVE, appears in **Figures 4A, B**. Notably, the project remained on track through early implementation, with 100% of planned activities progressing as expected until the first major macrosystem disruption in May 2023—the passage of the Parental Rights Bill. Although that legislative shift created tremendous challenges, we were able to pivot and obtain 30% response rates and usable survey data for the OPT.

However, beginning in early 2025, conditions shifted rapidly and cumulatively in ways that were beyond the control of the research team. These changes included significant and repeated administrative turnover within PHS. By the beginning of the first planned QED (spring, 2025), PHS was on its third superintendent since the project started (only two years prior). These disruptions extended beyond leadership changes to include shifting priorities, loss of institutional knowledge, and reduced capacity for coordination and support at the school level. Not only were administrative processes repeatedly restructured, but Project LIVE also lost key internal champions who had been instrumental in facilitating implementation.

As discrete events accumulated into patterns (culminating at the end of 2025) the challenges to implementation became increasingly untenable. Noteworthy is that shifting political norms created a “public safety crises” in immigrant and minoritized communities across the country (48–50). Within this broader context, PHS—and Project LIVE in particular—can be understood as casualties of intersecting policy, political, and institutional disruptions that extended far beyond the scope of the project itself.

## Lessons to inform future programming

Project LIVE highlights the reality that successful prevention efforts depend not only on intervention quality, community engagement, and methodological rigor, but also on broader structural conditions that shape implementation feasibility. Although the project was supported by federal funding, strong school and community partnerships, advisory boards, and a theoretically grounded curriculum, a series of macrosystem-level disruptions fundamentally altered the implementation environment. These experiences offer several important lessons for future prevention researchers and funding agencies.

First, prevention researchers should explicitly assess policy and political risk during study planning. Traditional community readiness assessments can provide useful information about local awareness, attitudes, and organizational capacity; however, they may be insufficient for large-scale prevention trials conducted in rapidly changing sociopolitical environments. Researchers should consider the potential impact of pending legislation, changes in consent requirements, administrative instability, immigration-related policies, funding uncertainties, and other structural factors that may influence recruitment, retention, implementation, and evaluation. While the specific events that affected Project LIVE could not have been predicted with certainty, greater attention to political and policy contexts may help identify vulnerabilities and support contingency planning before implementation begins.

Second, large-scale school-based prevention initiatives should establish multiple implementation champions and formalized institutional supports. Project LIVE relied heavily on relationships with district personnel who provided critical access to students, families, school administrators, and implementation resources. Although those partnerships remained strong throughout the project, repeated leadership turnover and organizational restructuring reduced institutional continuity and created challenges that extended beyond the capacity of individual collaborators to address. Future projects may benefit from establishing formal agreements, cross-training personnel, and cultivating support across multiple levels of leadership to reduce vulnerability to administrative transitions and shifting organizational priorities.

Third, recruitment challenges highlighted the importance of trust, community visibility, and local infrastructure in large-scale school-based prevention research. Project LIVE employed extensive recruitment strategies, including classroom visits, multilingual consent materials, phone calls, text messages, emails, attendance at community events, and neighborhood canvassing. The project also explored opportunities to partner with and hire individuals from within the local community to strengthen recruitment and community engagement efforts. However, these efforts were ultimately insufficient to overcome the broader structural barriers affecting participation. These experiences suggest that recruitment challenges in prevention research may not be resolved through increased outreach efforts alone when larger political, policy, and institutional forces are contributing to community fear, distrust, or disengagement. Future projects should carefully consider how broader sociopolitical contexts may shape community willingness to engage with research and should allocate substantial time and resources to building trust and recruitment infrastructure well before implementation begins.

Fourth, prevention trials should incorporate predefined decision points and contingency plans that allow for adaptation when implementation benchmarks become unattainable. Throughout Project LIVE, the research team repeatedly modified recruitment procedures, expanded eligible classes, developed new tracking systems, and introduced additional supports in an effort to preserve the original study design. While these adaptations were valuable, future projects may benefit from establishing clear thresholds for recruitment, retention, and implementation success, as well as predefined alternative strategies, implementation sites, or evaluation designs. Such planning may facilitate more efficient decision-making when external conditions substantially alter project feasibility.

Finally, funding agencies play a critical role in supporting prevention research when macrosystem disruptions fundamentally reshape implementation environments. Traditional funding structures often assume relatively stable conditions; however, Project LIVE demonstrates how policy changes, political events, institutional instability, and federal workforce disruptions can substantially alter research feasibility in ways that are outside the control of investigators and community partners. Importantly, our experience highlights the value of flexibility and collaboration between researchers and funders during periods of disruption. Throughout these challenges, CDC partners worked closely with the research team to identify feasible paths forward, including supporting adaptations to project activities and the exploration of alternative implementation and evaluation strategies. Such responsiveness allowed the project to preserve important scientific and practice-based contributions despite the inability to fully execute the original study design. Future prevention efforts may benefit from funding structures that continue to support adaptive designs, alternative implementation sites, modified evaluation approaches, and other pragmatic responses when major contextual disruptions occur.

Collectively, these lessons suggest that successful prevention science requires not only effective interventions and rigorous evaluation designs, but also explicit planning for the structural, political, and institutional conditions that shape whether those interventions can be implemented, sustained, and evaluated. Future efforts aimed at preventing CSEC and other forms of violence should incorporate these broader contextual considerations as core components of study design rather than treating them as external factors beyond the scope of implementation planning.

## Conclusion

In conclusion, this case study underscores a critical but underexamined reality in prevention science: the success of even well-designed, community-engaged interventions is contingent not only on program quality, but on the broader structural conditions in which they are implemented. Project LIVE was theoretically grounded, rigorously designed, and supported by strong community partnerships, yet was ultimately rendered infeasible due to intersecting macrosystem disruptions beyond the control of the research team. These findings challenge prevailing assumptions about the feasibility of school-based prevention trials and highlight the urgent need for research designs, funding structures, and policy environments that are responsive to real-world conditions. Moving forward, prevention science must expand its focus beyond questions of effectiveness to include the conditions under which effective interventions can be implemented, sustained, and evaluated, particularly in communities most impacted by structural inequities.

## Data Availability

The raw data supporting the conclusions of this article will be made available by the authors, without undue reservation.

## Footnotes

1 Project LIVE was one of four projects receiving the 2026 Iowa Outstanding Anti-trafficking Service Award.

2 READY stands for **R**eport anything dangerous; **E**nd the communication; **A**sk for help; **D**on’t engage; and **Y**our safety first (33).

3 There are increased calls to empower bystanders to prevent CSEC (34); shifting school norms to be intolerant of CSEC and gender-based violence may lead to reductions in sexual exploitation (35).

4 These are the most recent numbers reported, as of manuscript submission.
